# Supplementary material for: Slow oscillation-sleep spindle coupling is associated with fear extinction retention in trauma-exposed individuals
Source: bioRxiv. 2025 Jan 28:2025.01.27.634866. Preprint. [Version 1] doi: 10.1101/2025.01.27.634866 (PMC11838212; doi:10.1101/2025.01.27.634866)
Supplement: 1 [file NIHPP2025.01.27.634866V1-supplement-1.pdf]

bioRxiv preprint doi: https://doi.org/10.1101/2025.01.27.634866; this version posted January 28, 2025. The copyright holder for this preprint (which was not certified by peer review) is the author/funder, who has granted bioRxiv a license to display the preprint in perpetuity. It is made available under aCC-BY 4.0 International license.

Supplementary Materials

Skin conductance responses

SCRs were calculated for each trial as the mean skin conductance level in μS during the last two seconds of study context presentation subtracted from the maximum skin conductance level during the six seconds of colored lamp presentation. SCRs were square-root transformed, and recoded to zero in cases where the untransformed SCR was negative (1). Non-conditioning participants were excluded and were defined as those who exhibited less than 2 non-square-root transformed SCR responses to either of the two CS+s (in any combination) that were equal to or exceeding 0.05 μS during the Fear Conditioning phase (2). The first presentations of CS+1 and CS+2 during Fear Conditioning were excluded from analyses because their pairing with the unconditioned stimulus (shock) had not yet occurred. Similarly, these first presentations were not considered for the requirement of 2 non-transformed SCRs ≥ 0.05 μS.

Extinction recall and generalization indices

The extinction retention index (ERI) was calculated as each participant's average SCR to the first four CS+E trials of the extinction recall phase divided by their largest SCR to a CS+ trial during conditioning and multiplied by 100, yielding a percentage of the maximal conditioned fear that had been retained (2,3). Again, the first presentations of CS+1 and CS+2 during Fear Conditioning were excluded from use as the largest SCR to a CS+. Lower ERI indicates better extinction retention. The extinction generalisation index (EGI) was calculated by subtracting the average of the first four CS+E trials of extinction recall from the average of the first four CS+U trials of extinction recall, dividing this difference by the largest SCR to a CS+ trial during fear conditioning and multiplied by 100. This yields a percentage of the maximum conditioned fear represented by the retained difference in response to an unextinguished vs extinguished CS+ (4).

Statistical analysis

Group differences in SCRs were examined via a linear mixed effects model with the factors Group (TEC, PTSD), Stimulus type (either CS+E, CS- for extinction recall, or CS+E, CS+U for extinction generalisation), Trial (first, second, third, fourth) and their interactions entered as fixed effects, and participant entered as a random effect.

Results

Behaviour

When comparing CS+E to CS-, there was a significant interaction between Stimulus type and Trial (F (1,614)=4.38, p=.036; **Figure S1**). On the first trial, SCRs to the CS+E were significantly higher compared to the CS- (p<.001). There was no difference between the CS+E and CS+U (F (1,624)=0.27, p=.61). No main effects or interactions involving group were observed (ps > .52).

bioRxiv preprint doi: https://doi.org/10.1101/2025.01.27.634866; this version posted January 28, 2025. The copyright holder for this preprint (which was not certified by peer review) is the author/funder, who has granted bioRxiv a license to display the preprint in perpetuity. It is made available under aCC-BY 4.0 International license.

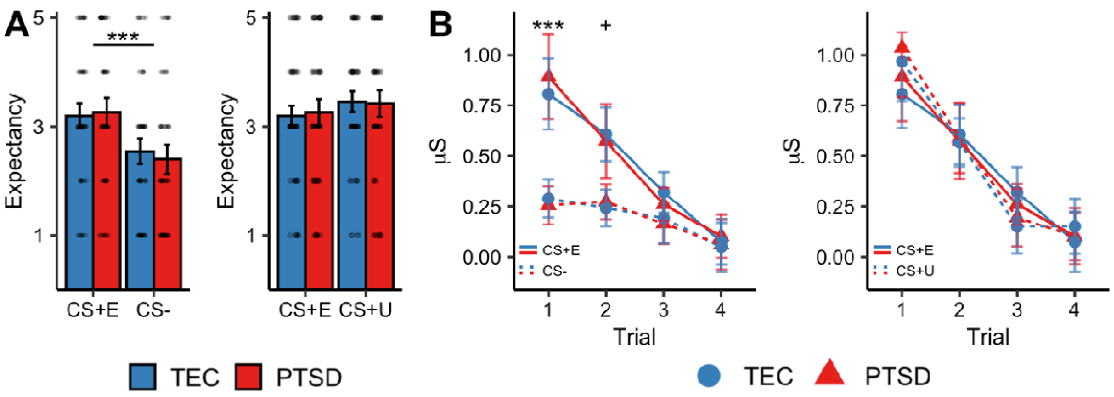

Figure S1. Behavioural and SCR results. **A** – Expectancy ratings to the first trial of each CS type. Expectancy ratings to the CS+E stimulus was significantly higher than the CS- (left panel), and no different to the CS+U (right panel). **B** – SCR responses to the first four trials of each CS type. Higher SCR responses were found to the first CS+E trial compared to the first CS- (left panel). No differences were found between CS+E and CS+U trials (right panel). *** = $p < .001$, + = $p < .10$. All error bars show the 95% confidence intervals around the mean.

Associations between SO-spindle coupling and SCR measures of extinction

We ran exploratory regression analyses examining relationships between SO-spindle coupling and SCR-derived measures of extinction recall and generalisation. No significant associations were found (ps>.13).

References

1. Lonsdorf, T. B., Menz, M. M., Andreatta, M., Fullana, M. A., Golkar, A., Haaker, J., Heitland, I., Hermann, A., Kuhn, M., Kruse, O., Meir Drexler, S., Meulders, A., Nees, F., Pittig, A., Richter, J., Römer, S., Shiban, Y., Schmitz, A., Straube, B., … Merz, C. J. (2017). Don't fear 'fear conditioning': Methodological considerations for the design and analysis of studies on human fear acquisition, extinction, and return of fear. *Neuroscience & Biobehavioral Reviews*, *77*, 247–285. https://doi.org/10.1016/j.neubiorev.2017.02.026
2. Bottary, R., Seo, J., Daffre, C., Gazecki, S., Moore, K. N., Kopotiyenko, K., Dominguez, J. P., Gannon, K., Lasko, N. B., Roth, B., Milad, M. R., & Pace-Schott, E. F. (2020). Fear extinction memory is negatively associated with REM sleep in insomnia disorder. *Sleep*, *43*(7). https://doi.org/10.1093/sleep/zsaa007
3. Pace-Schott, E. F., Spencer, R. M. C., Vijayakumar, S., Ahmed, N. A. K., Verga, P. W., Orr, S. P., Pitman, R. K., & Milad, M. R. (2013). Extinction of conditioned fear is better learned and recalled in the morning than in the evening. *Journal of Psychiatric Research*, *47*(11), 1776–1784. https://doi.org/10.1016/j.jpsychires.2013.07.027
4. Pace-Schott, E. F., Milad, M. R., Orr, S. P., Rauch, S. L., Stickgold, R., & Pitman, R. K. (2009). Sleep Promotes Generalization of Extinction of Conditioned Fear. *Sleep*, *32*(1), 19–26. https://doi.org/10.5665/sleep/32.1.19
